# Absence of Part of the Diaphragm

**Published:** 1881-12

**Authors:** 


### ABSENCE OF PART OF THE DIAPHRAGM.

Polaillon (Annales de Gynæcologie, April, 1881), reports two cases of congenital absence of part of the diaphragm with consequent visceral displacement; death occurred soon after birth. The symptoms present were thoracic respiration only, cardiac displacement, great dyspnœa and intra-thoracic borborygmi. Death generally results from pulmonary compression, although cardiac involvement may aggravate this if the malformation be on the left side, as has been found to be the case in twenty-three out of the thirty-seven instances thus far reported.

